# Prophenoloxidase Activation Is Required for Survival to Microbial Infections in *Drosophila*

**DOI:** 10.1371/journal.ppat.1004067

**Published:** 2014-05-01

**Authors:** Olivier Binggeli, Claudine Neyen, Mickael Poidevin, Bruno Lemaitre

**Affiliations:** 1 Global Health Institute, School of Life Sciences, Ecole Polytechnique Fédérale de Lausanne (EPFL), Lausanne, Switzerland; 2 Centre de Génétique Moléculaire (CGM), CNRS, Gif-sur-Yvette, France; Stanford University, United States of America

## Abstract

The melanization reaction is a major immune response in Arthropods and involves the rapid synthesis of melanin at the site of infection and injury. A key enzyme in the melanization process is phenoloxidase (PO), which catalyzes the oxidation of phenols to quinones, which subsequently polymerize into melanin. The *Drosophila* genome encodes three POs, which are primarily produced as zymogens or prophenoloxidases (PPO). Two of them, PPO1 and PPO2, are produced by crystal cells. Here we have generated flies carrying deletions in *PPO1* and *PPO2*. By analyzing these mutations alone and in combination, we clarify the functions of both PPOs in humoral melanization. Our study shows that PPO1 and PPO2 are responsible for all the PO activity in the hemolymph. While PPO1 is involved in the rapid early delivery of PO activity, PPO2 is accumulated in the crystals of crystal cells and provides a storage form that can be deployed in a later phase. Our study also reveals an important role for PPO1 and PPO2 in the survival to infection with Gram-positive bacteria and fungi, underlining the importance of melanization in insect host defense.

## Author Summary

The melanization reaction is a major immune response in Arthropods and involves the rapid synthesis of a black pigment, melanin, at the site of infection and injury. Melanization requires the activation of proPhenoloxidase, an enzyme that catalyzes the oxidation of phenols to quinones, which polymerize to melanin. The *Drosophila* genome contains three genes encoding prophenoloxidases (PPO). In this paper, we have generated flies carrying deletions in the *PPO1* and *PPO2* genes. By analyzing these mutations alone and in combination, we clarify the functions of both prophenoloxidases in humoral melanization. We report that PPO2 composes most of the crystals found in crystal cells, a specific hemocyte cell type. Although PPO1 and PPO2 both contribute to phenoloxidase activity in the insect blood, these PPOs are not fully redundant. Our study shows that PPO1 is involved in the rapid delivery of phenoloxidase activity when required, while PPO2 provides a storage form that can be deployed in a second phase. Some controversy exists in the *Drosophila* field about the importance of melanization in the *Drosophila* host defense. Our study demonstrates the important role of PPO1 and PPO2 in the survival to infection with both Gram-positive bacteria and fungi, underlining the importance of melanization in insect immunity.

## Introduction

One of the most immediate immune responses in arthropods is the melanization reaction [1], [2]. It involves the rapid synthesis of melanin at the site of infection or injury in order to contain a microbial pathogen as well as to facilitate wound healing. A key enzyme in melanin biosynthesis is phenoloxidase (PO), which catalyzes the oxidation of phenols to quinones, which subsequently polymerize into melanin. PO is usually synthesized as an inactive zymogen called proPO (PPO), which is cleaved to generate active PO as a result of proteolytic cascade activation. Several roles have been ascribed to the melanization reaction in insects [3], [4]. PO activity contributes to wound healing by forming a scab at the epithelial site of injury. By-products of PO activity are reactive oxygen species (ROS), which are thought to contribute to the killing of microbes and pathogens. Finally, melanization participates in the encapsulation reaction against parasites. Deposition of melanin on the parasite forms a physical barrier, allowing the localized and confined production of toxic compounds while ensuring the protection of the host.

Despite extensive genetic studies of the *Drosophila* immune response, the melanization reaction remains one of its less characterized facets. The *Drosophila* genome encodes three PPOs. Two of them, PPO1 and PPO2, are found in the crystal cells and possibly in the hemolymph at the larval stage. Crystal cells represent 5% of the hemocyte (blood cells) population of larva [5]–[7]. Upon injury, crystal cells rupture and release PPOs into the hemolymph where they are activated by serine proteases [8]. Although PPO1 and PPO2 are found in the hemolymph compartment in adults, their precise sites of synthesis have not been characterized. Notably, the presence of crystal cells has not yet been established in adult flies [7]. Some reports have suggested that PPO3 is expressed in crystal cells [9] while others suggest an expression in lamellocytes [10], [11]. Lamellocytes are larval hemocytes involved in the encapsulation of parasites such as parasitoid wasps. The production of PPO3 in lamellocytes points to a role of this enzyme in capsule formation. While PPO3 is produced under an active form, both PPO1 and 2 require a proteolytic cleavage to be activated [12]. The cleavage of PPO1 is mediated by a clip-domain serine protease (SP) named Hayan [13]. Hayan also exists as an inactive zymogen that is itself stimulated through a stepwise process involving other serine proteases, whose activities are all controlled by protease inhibitors named serpins. Two clip-domain SPs, MP1 and the crystal cell-specific Sp7 (also referred to as MP2 and PAE), and several serpins have been implicated in the melanization cascade upstream of Hayan [14]–[21]. Inactivation of MP1 or MP2 reduces the level of PO activity after immune challenge, while excessive melanization is usually observed in Serpin-deficient mutants. Studies in other insect species indicate that the SP cascades upstream of PPO are triggered by injury or by pathogen recognition receptors detecting microbial ligands, such as peptidoglycan or β(1,3)-glucan [22]–[24]. Accordingly, full PO activation in *Drosophila* also requires triggering of Toll pathway-specific pattern-recognition receptors by Gram- positive bacteria (via GNBP1 and PGRP-SA) or fungi (via GNBP3) [25]. These receptors are found in the hemolymph of flies and probably activate PO by SPs distinct from those triggering Toll activation by Spätzle. The melanization cascade is also regulated at the transcriptional level since many transcripts encoding related enzymes, SPs or serpins, are upregulated in the fat body by the Toll and Imd pathways in response to infection [14], [15].

Several studies have analyzed the contribution of melanization to survival of flies with variable results. These studies have used fly stocks carrying either mutations that abolish (almost) all PO hemolymphatic activity (using the uncharacterized mutation *Black cells* or *Hayan* ^1^) or reduce it (*Sp7* mutation or *in vivo RNAi* against *Sp7* or *MP1*) [13], [16]–[18], [26]. It was observed that both *Black cells* and *Hayan* ^1^ flies exhibit increased susceptibility to large injury [11], [27]. *Black cells* but not *Sp7* flies were shown to be susceptible to the Gram-positive bacterium *Enterococcus faecalis* [17], [26]. *Black cells* and *Sp7 RNAi* flies but not the *Sp7* mutants were shown to be susceptible to the entomopathogenic fungus *Beauveria* bassiana [14], [17], [18]. An extensive study also reports increased susceptibility of the *Sp7* mutants to the Gram-negative bacteria *Salmonella typhimurium* and Gram-positive bacteria *Listeria monocytogenes* and *Staphylococcus aureus* [26];although a susceptibility of the *Sp7* mutant to *S. aureus* was not observed in another report [17].

These studies show that our knowledge of the function and regulation of POs in *Drosophila* is still scarce. One reason is that most studies have used a top-down approach focusing on the most upstream steps of the melanization reaction, which appears ramified. In this study, we have focused on the downstream step of the melanization cascade by generating flies carrying deletions for *PPO1* and/or *PPO2*. By analyzing these mutations alone and in combination, we characterize the functions of both PPOs in the melanization reaction. Our study also confirms and extends previous studies showing an essential role of this reaction against some Gram-positive bacteria and fungi and wasp encapsulation.

## Results

### A gene-deletion strategy to study Phenoloxidase function

**Figure 1:**
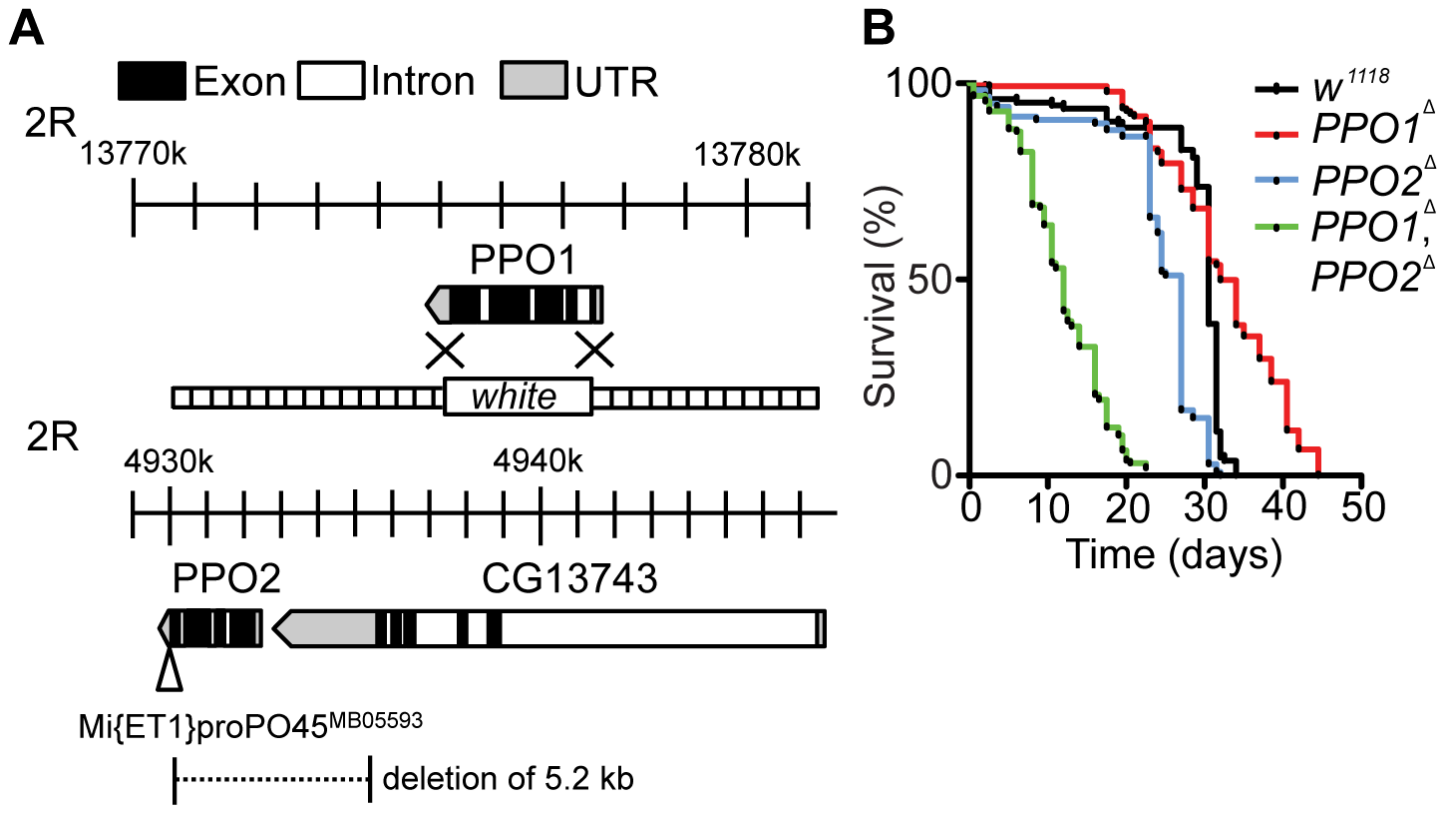
Generation and phenotypic characterization of *PPO1* and *PPO2* deletion mutants. (**A**) Schematic representation of *PPO1* ^Δ^ and *PPO2* ^Δ^ deletions. The gene map was adapted from FlyBase and includes *PPO1* (top) and *PPO2* with its neighboring gene *CG13743* (bottom). The *PPO2* mutant was generated after the mobilization of the transposable element *Mi{ET1}PPO2^ MB05593^* inserted in the 3′ end of *PPO2*. The imprecise excision deleted a fragment of 5.2 kb including the 3′ non-coding sequence of the neighboring gene *CG13743*. *PPO1* ^Δ^ mutant flies were generated by homologous recombination of *PPO1* with the *w* gene. The flanking sequences used for recombination (dotted lines) are indicated. (**B**) Lifespan analysis of unchallenged flies reveals an increased mortality of *PPO1* ^Δ^, *PPO2* ^Δ^ double mutants (***, p<0.0005). Each survival curve corresponds to one experiment of 3 samples with 20 flies each. *p* values were calculated using the Log-rank test.

Through homologous recombination, we replaced the entire *PPO1* locus (Chromosome II, position 55C) with a copy of the *white* gene (referred to as *PPO1* ^Δ^) (**Fig. 1A**). We also generated a deletion of 5.2 kb removing the entire coding sequence of *PPO2* (Chromosome II, position 45A1) by imprecise excision of a *Minos* transposon (referred to as *PPO2* ^Δ^). The latter deletion also removes 2 kb of the 3′ non-coding region of the *CG13743* gene, which encodes an amino-acid transporter (**Fig. 1A**). Both *PPO* deficient lines were backcrossed five times into either *w^1118^* or OregonR to homogenize the genetic background. In this study, we mainly used *w^1118^*, *PPO* deficient flies but obtained similar results with lines backcrossed in OregonR. To explore possible redundancy between the two genes, a double mutant (*PPO1* ^Δ^, *PPO2* ^Δ^) was produced by meiotic recombination. The absence of *PPO1* and *PPO2* transcripts in the respective mutants was confirmed by RT-qPCR (**Fig. S1A**). We also verified that the *PPO2* deletion did not affect the expression of the flanking *CG13743* gene (**Fig. S1B**). Both *PPO1* ^Δ^ and *PPO2* ^Δ^ mutants and the *PPO1* ^Δ^, *PPO2* ^Δ^ double mutants are viable and do not exhibit any overt developmental or pigmentation defect (**Fig. S1C**). We noticed that the *PPO1* ^Δ^, *PPO2* ^Δ^ double mutants have a significantly shorter lifespan (half-life = 24 days, *p*<0.0001 by Log rank test) than the single mutants and wild-type (**Fig. 1B**). A similar trend towards reduced lifespan was observed when *PPO1* ^Δ^, *PPO2* ^Δ^ double mutant flies were raised in germ-free conditions (**Fig. S1D**), suggesting that early lethality is not due to infection. In this study, we focused on the role of PPOs in the defense against microbial pathogens in larvae and adults.

### PPO2 is stored in the crystals of crystal cells

**Figure 2:**
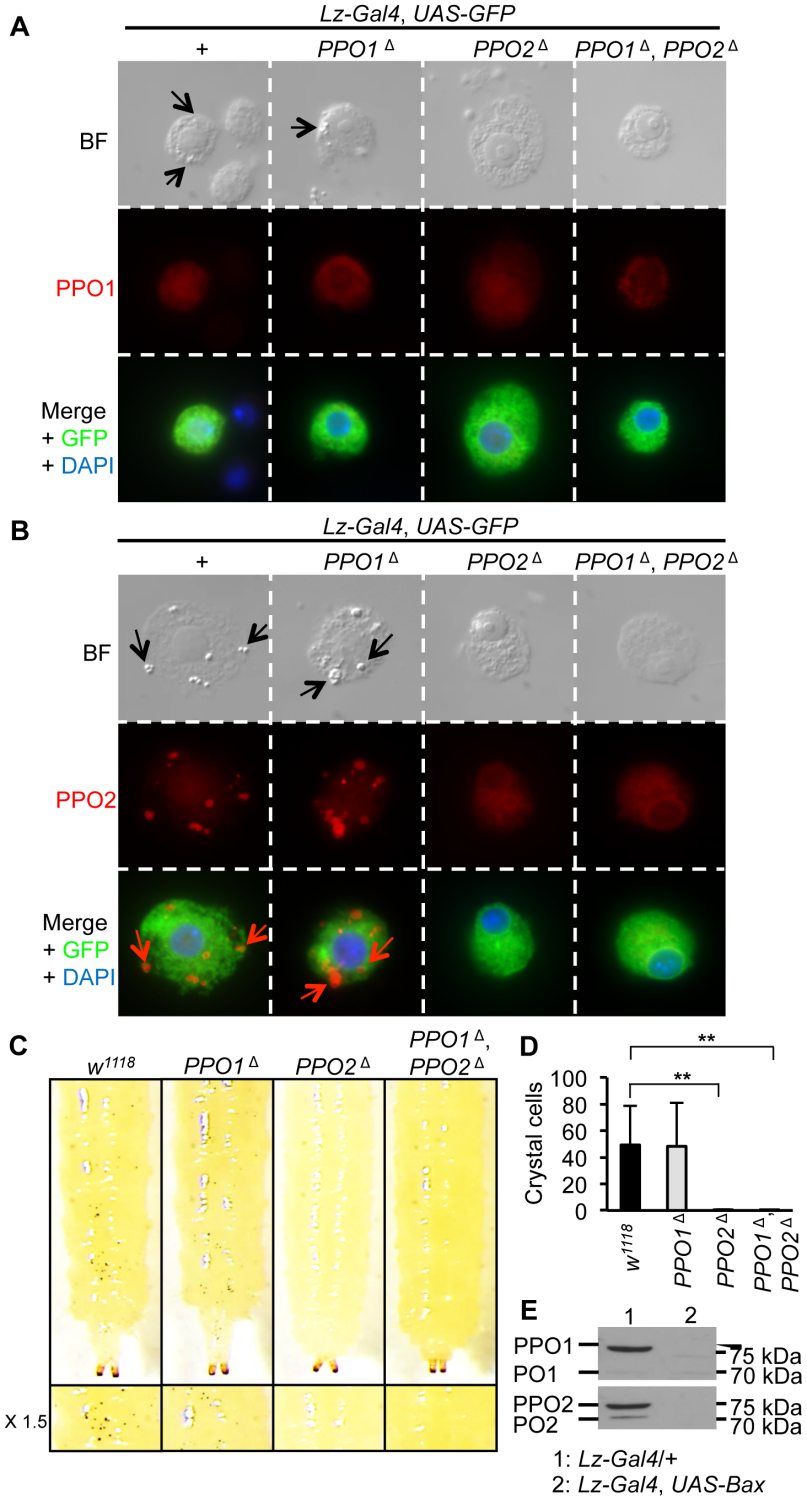
PPO2 forms the crystals of crystal cells. (**A**) Bright field (BF) and fluorescence micrographs of crystal cells of L3 larvae expressing a *Lz-Gal4*, *UAS-GFP* construct combined with the *PPO* mutations (top panel). Arrows indicate crystals within crystal cells. Immunostaining with antibodies against PPO1 (red), or GFP (green) reveals that PPO1 antibody does not detect PPO1 in the crystals of crystal cells (medium and bottom panels). (**B**) Immunostaining with antibodies against PPO2 (red), or GFP (green) shows that PPO2 composes the crystals of crystal cells. Specificity of the antibody was confirmed by the absence of signal in crystal cells from *PPO2* ^Δ^ and *PPO1* ^Δ^, *PPO2* ^Δ^ mutant larvae (medium and bottom panels). Arrows indicate crystals within crystal cells. (**C**) No melanization of crystal cells was observed in *PPO2* ^Δ^ and *PPO1* ^Δ^, *PPO2* ^Δ^ double mutant larvae after spontaneous activation of the PPO by incubating larvae at 65°C for 10 minutes. (**D**) Number of melanized crystal cells found in the posterior region of L3 larvae after heating. No melanized crystal cells were found in *PPO2* ^Δ^ and *PPO1* ^Δ^, *PPO2* ^Δ^ double mutant larvae. Data were analyzed by t test and values represent the mean ± s.e. of at least 10 different larvae per genotype. (**E**) PPO1and PPO2 are not detected upon crystal cell depletion. Hemolymph samples were derived from unchallenged F1 progeny of *Lz-Gal4*, *UAS-GFP* crossed with *UAS-Bax*/*CyO-GFP* larvae. A representative Western Blot analysis using *Drosophila* anti-PO1 and anti-PO2 antibodies is shown.

In *Drosophila* larvae, PPO1 and 2 are synthetized by specific hemocytes termed crystal cells [5], [28]. Massive release of PPO involves both rupture of crystal cells and dissolution of the crystals [8]. It has long been assumed but never really demonstrated that the crystals within crystal cells are composed of PPO. To clarify this point, we analyzed the morphology of crystal cells in third instar *PPO* mutant larvae. As crystal cells make up only 5% of all larval hemocytes [6], [7], we used the crystal cell marker *lozenge* (*Lz-Gal4*, *UAS-GFP*) to identify crystal cells in hemolymph preparations of *PPO* mutants [29]. **Fig. 2A–B** shows that crystals are present in both wild-type and *PPO1* crystal cells (arrows in bright field panels) but in neither *PPO2* ^Δ^ nor *PPO1* ^Δ^, *PPO2* ^Δ^ double mutants. This indicates that PPO2 is either the major component of the crystals or that it is required for their formation. Heating larvae at 65°C for 10 min induces the spontaneous activation of PPO within the crystal cells [5]. As a consequence of this treatment, crystal cells are easily visualized through the cuticle as black dots. We used this method to analyze how PPO1 and PPO2 contribute to crystal cell melanization. Consistent with our observations, *PPO2* ^Δ^ mutant larvae and *PPO1* ^Δ^, *PPO2* ^Δ^ double mutants did not show any melanized dots upon heating while *PPO1* behaved like wild-type (**Fig. 2C** and **Fig. 2D**). PPO2- specific antibody staining co-localizes with crystals, demonstrating that crystals are indeed composed of PPO2 (**Fig. 2B**). In contrast, no overt signal could be detected in crystal cells using an antibody that recognizes the native form of PPO1 (**Fig. 2A**). Nevertheless, we cannot exclude that the anti-PPO1 antibody does not work in immunostaining experiments or that a low amount PPO1 is still present in the crystals but hidden by PPO2. To confirm that crystal cells are the sole source of PPO1 and PPO2 in larvae, we analyzed by Western blot the presence of the PPOs in hemolymph samples of larvae with genetic ablation of crystal cells (genotype: *Lz-Gal4, UAS-Bax*). Neither PPO1 nor PPO2 were observed in *Lz-Gal4, UAS-Bax* larvae confirming that the two PPOs are indeed produced by crystal cells (**Fig. 2E**). Collectively, our study shows that PPO2 is stored in crystal cells while PPO1 is either a minor component of the crystal or most probably released from crystal cells into the hemolymph.

### PO1 and PO2 both contribute to injury-mediated melanization in larvae and adults

**Figure 3:**
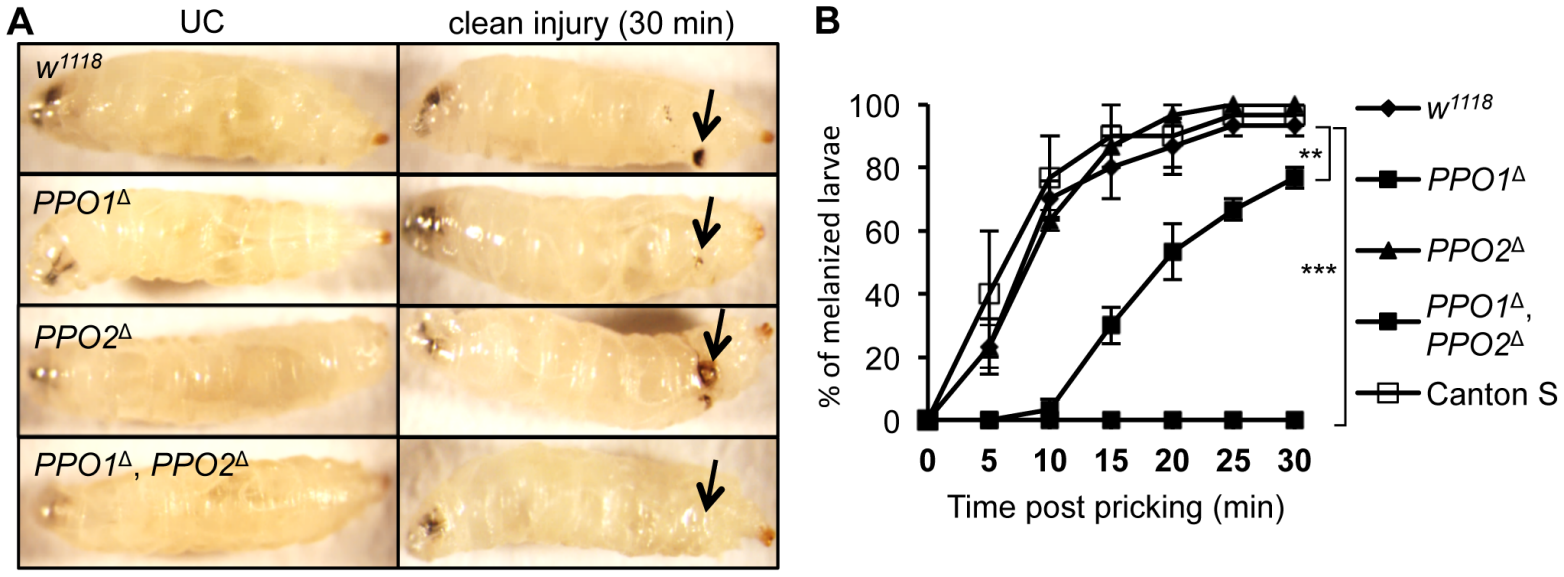
Both PPO1 and PPO2 contribute to injury related melanization in larvae. (**A**) Melanization of larvae after clean injury is abolished in the simultaneous absence of PPO1 and PPO2. A reduced melanization spot was observed in *PPO1* ^Δ^ mutants. *PPO2* ^Δ^ mutant larvae displayed a more intense melanization at the wound site when compared to wild-type. Arrows indicate the pricking site. Larvae were wounded with a tungsten needle and blackening of the wound was recorded 30 minutes later. A representative picture is shown for each genotype. (**B**) *PPO1* ^Δ^ larvae have a defect in melanization rate upon pricking compared to wild-type and *PPO2* mutant larvae. *Cantons^S^* larvae were used as an additional wild-type control. Larvae were wounded with a tungsten needle and the presence of a blackening wound was recorded every 5 minutes up to 30 minutes. Data were analyzed using the Log rank test and values represent the mean±s.e. of at least three independent experiments of 10 larvae each.

Needle injury to wild-type larvae or adults induces a melanization reaction at the wounding site, whose extent is usually proportional to the injury size. This blackening reaction results from *de novo* synthesis of melanin by PO and is also enhanced by the presence of microbial products [13]. We investigated the role of PPO1 and PPO2 in this process by observing the melanization site 30 min after clean injury in larvae (**Fig. 3A**). While a reduced melanization spot was observed in *PPO1* ^Δ^ mutants, *PPO2* ^Δ^ mutant larvae displayed a more intense melanization at the wound site compared to wild-type. No melanization spot was observed in injured *PPO1* ^Δ^, *PPO2* ^Δ^ double mutants. This indicates that both PPO1 and PPO2 contribute to injury-mediated melanization. Our results also suggest that the presence of PPO2 has a slight inhibitory effect on PO activity. A kinetic analysis shows that melanization at the wound site is delayed in *PPO1* ^Δ^ mutants when compared to wild-type (**Fig. 3B**). This reveals a sequential requirement of the two PPOs: PPO1 provides an immediate source of PO while PPO2 becomes available later.

**Figure 4:**
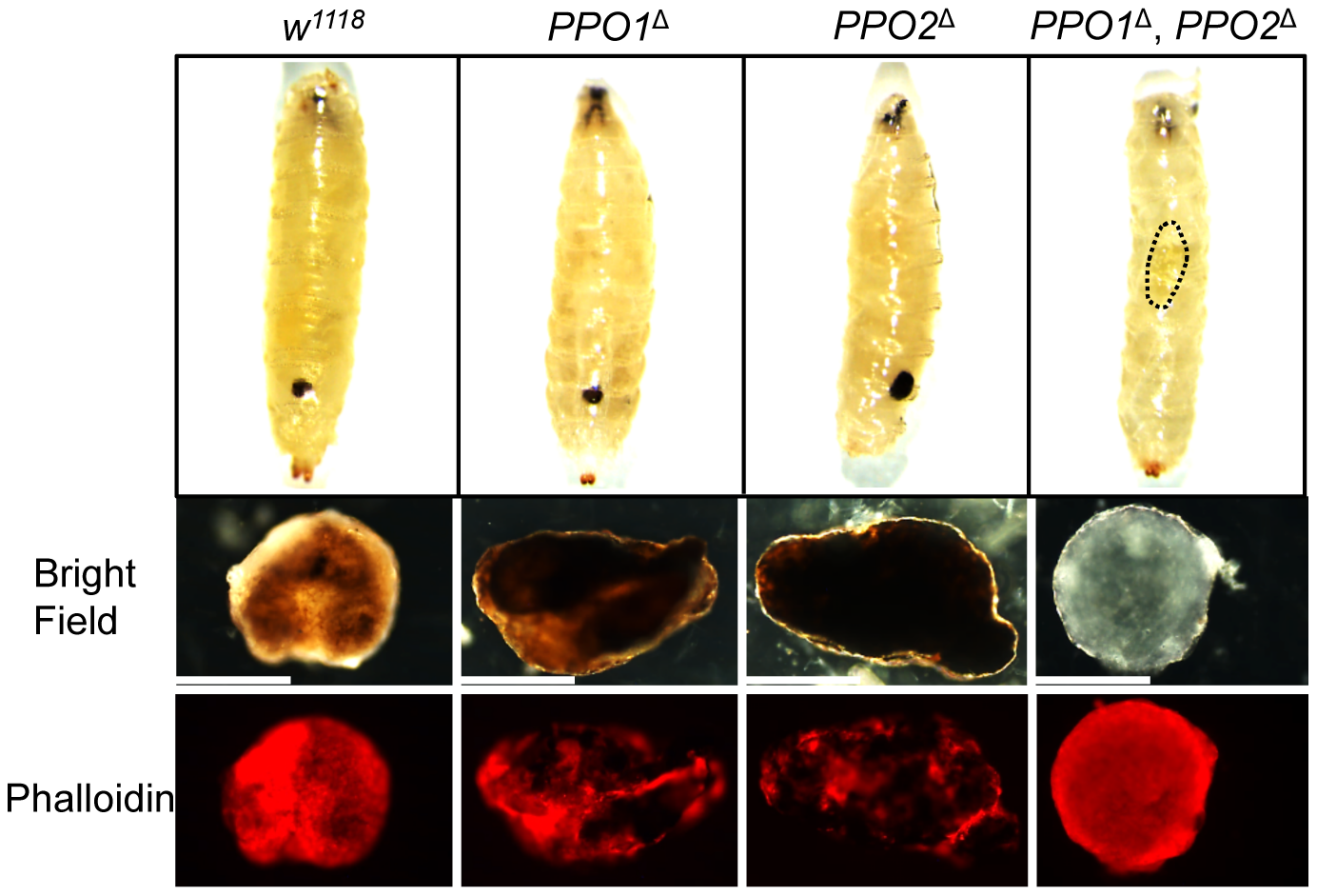
Both PPO1 and PPO2 contribute to melanization of wasp capsule. Top panels: representative photos showing infected eggs of *A. tabida* parasitoid wasp were not melanized in the *PPO1* ^Δ^, *PPO2* ^Δ^ mutant larvae. Note that *A. tabida* larvae escape the capsule and develop better in the double-mutant compared to single mutants and wild-type (the position of the larva is indicated with dashed dots). Lower panels: Light micrographs showing melanin deposited on the surface of the eggs of *A. tabida* removed from the hemocoel of wild-type and *PPO* mutant larvae at 6 days post-infection. Phalloidin staining reveals the presence of lamellocytes around the egg of wild-type and *PPO* mutant larvae. Bars  = 300 µm.

We next investigated the role of PPO in the encapsulation of parasites. Wild-type and *PPO* mutant second instar larvae were infected with *Asobara tabida*, a parasitoid wasp that is usually encapsulated in *Drosophila melanogaster* [30]. **Fig. 4** shows that *A. tabida* is encapsulated in the wild-type and single mutants but not in the *PPO1* ^Δ^, *PPO2* ^Δ^ double mutants. Staining with phalloidin reveals that the wasp egg is still covered by lamellocytes in the *PPO1* ^Δ^, *PPO2* ^Δ^ mutant. This confirms that melanization is not required for encapsulation by lamellocytes consistent with a previous report [31].

**Figure 5:**
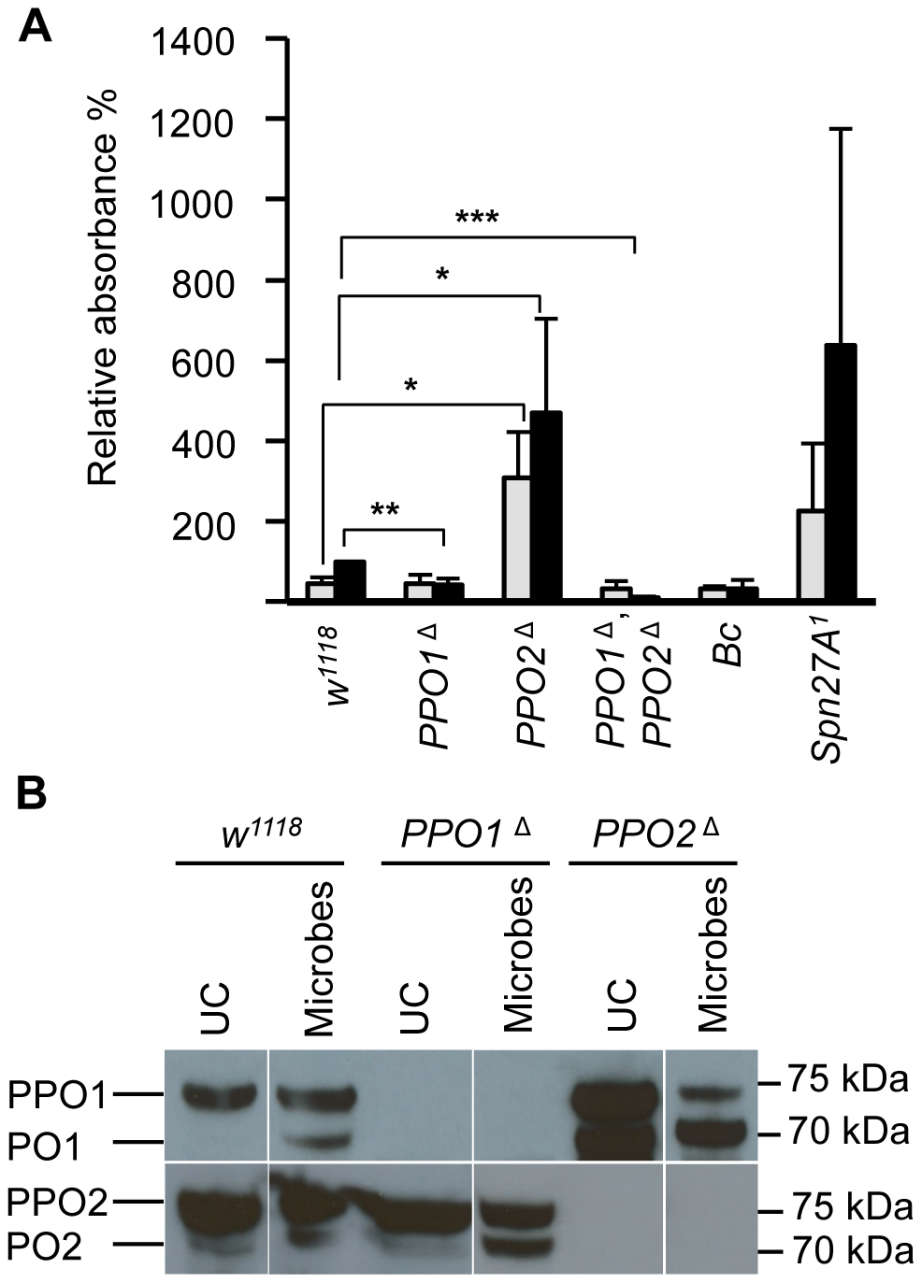
Increase of PO activity in absence of PPO2. (**A**) Hemolymph PO activity in unchallenged and wounded adult flies. Spontaneous PO activity is abolished in *PPO1* ^Δ^, *PPO2* ^Δ^ double mutant flies (***, p<0.0005) and strongly reduced in *PPO1* ^Δ^ mutants (**, p<0.005) compared to wild-type. In *PPO2* ^Δ^ deficient flies, a stronger PO activity is observed even in the absence of wounding (*, p<0.05). Adult flies were punctured and 3 h later their hemolymph was examined for PO activity. Hemolymph of wounded *Bc* flies and unchallenged *Spn27A^1^* flies was used as control. Data were analyzed by t test and values represent the mean±s.e. of three independent experiments. (**B**) A representative Western Blot analysis using *Drosophila* anti-PPO1 (top) and anti-PPO2 (bottom) antibodies. Hemolymph samples were collected from adult flies subjected to injury contaminated with a mixture of Gram-positive *Micrococcus luteus* and Gram-negative *Erwinia carotovora carotovora 15* (*E. carotovora*) bacteria. Absence of PPO1 and PPO2 in the respective single and double mutant flies is confirmed. Of note, higher levels of the mature form of PPO1 were observed in *PPO2* ^Δ^ mutants consistent with the notion that PPO2 negatively impacts PPO1. Hemolymph was extracted from flies 3 h post wounding.

We next focused our attention on the role of PPO1 and PPO2 in adults. As observed in larvae, a reduced melanization spot was observed in *PPO1* ^Δ^ flies while *PPO2* ^Δ^ flies displayed a more intense melanization at the wound site compared to wild- type. No melanization spot was observed in injured *PPO1* ^Δ^, *PPO2* ^Δ^ flies whatever the microbe used (**Fig S2A, S2B, data not shown**). We next measured enzymatic PO activity with an L-DOPA assay in adult hemolymph samples from wild-type and *PPO* mutants. In these experiments, we also included as controls hemolymph samples from *Black cells* mutant flies which carry an uncharacterized mutation affecting PO activity [5], and from *Spn27A^1^* deficient flies that exhibit constitutive PO activity [14], [15]. Unchallenged wild-type flies showed only a low level of PO activity (**Fig. 5A**). 4 h after septic injury PO activity increased as previously reported [17]. No significant PO activity was detected in the hemolymph of naive or infected *PPO1* ^Δ^ single and *PPO1* ^Δ^, *PPO2* ^Δ^ double mutants. *PPO2* ^Δ^ mutants however showed a significantly higher PO activity even in the absence of challenge, which further increased after septic injury. This is consistent with the apparent negative regulatory role of PPO2 observed in injured larvae.

As mentioned above, PPO1 and PPO2 are synthesized as inactive zymogens called prophenoloxidases (PPOs), which are cleaved by SP activity to generate the active form. To determine the relative protein levels of both circulating PPOs and POs in *PPO* deficient flies, we performed Western blot analyses with hemolymph extracts using anti- PPO1 and anti-PPO2 antisera [13]. The Western blot confirmed the absence of PPOs in the respective mutants. In control wild type flies, PPO1 was detected as a single band of ∼75 kDa, which correlates with its expected molecular weight [13], [17] (**Fig. 5B**). Septic injury induces the appearance of a shorter band of ∼70 kDa corresponding to the mature form. In the absence of injury, PPO2 was detected as a doublet: a major band of ∼75 kDa corresponding to PPO and a minor band of ∼70 kDa corresponding to the mature form (**Fig. 5B**). Septic injury also increased the mature form of PPO2. Consistent with the notion that PPO2 exhibits a higher PO activity, *PPO2* ^Δ^ mutants showed higher total amounts of PPO1 and increased levels of the mature form of PPO1 in both naïve and injured flies. Since we did not see a compensatory up-regulation of *PPO1* mRNA in *PPO2* ^Δ^ mutant flies (**Fig. S1A**), PPO1 is likely regulated at post-translational level by PPO2. Collectively, our study shows that both PPO1 and PPO2 contribute to the melanization observed in the hemolymph or at the wound site in flies upon injury.

### Epistatic relationships between PPO1, PPO2 and Serpin 27A

**Figure 6:**
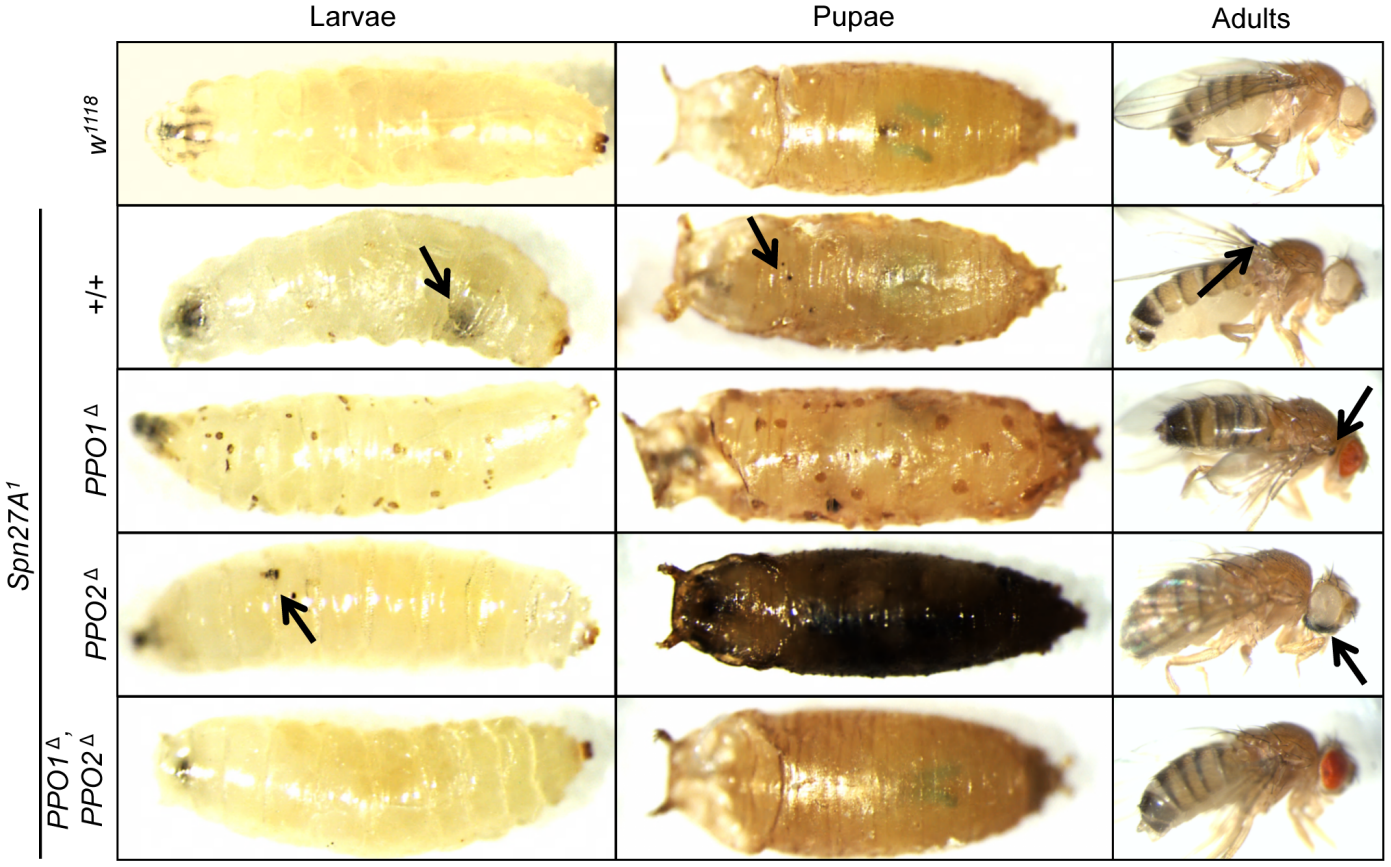
Epistatic relationships between Serpin 27A and PPOs. Pictures of larvae (left panel), pupae (center panel) and adults (right panel) were taken in the absence of wounding. *Spn27A^1^* mutants exhibit spontaneous melanization (black arrows) which is suppressed in a *PPO1* ^Δ^ *PPO2* ^Δ^ double mutant background. This uncontrolled melanization is not completely abolished in *PPO1* ^Δ^, *Spn27A^1^* mutant flies (note the melanized spot indicated by the arrow). A distinctive pattern of melanized spots under the epidermis was observed in *Spn27A^1^, PPO1* ^Δ^ larvae and pupae. The *Spn27A^1^* melanization was stronger in the absence of *PPO2* with pupae turning black and adults presenting black spots and wing defects (not shown).

Spn27A negatively regulates the hemolymph PPO-activating cascade via inhibition of a yet unidentified SP upstream of Hayan [13]–[15], [32]. Mutations in *Spn27A* cause lethality and ectopic melanization. We therefore investigated the role of PPO1 and 2 in the *Spn27A^1^* phenotype by recombining the null *Spn27A^1^* mutation with *PPO1* ^Δ^ and/or *PPO2* ^Δ^. The spontaneous melanization found in *Spn27A^1^* deficient animals is suppressed by the combined absence of *PPO1* and *PPO2* (**Fig. 6**). The lethality induced by *Spn27A* *^1^* deficiency is also largely but not completely rescued in the absence of both PPOs (**Fig. S3**). We further observed that some 50% of the *Spn27A^1^*, *PPO1* ^Δ^ larvae exhibit a characteristic pattern of melanization spots under the epidermis, which could correspond to the attachment sites of sessile hemocytes [6], [33], [34]. Interestingly, the *Spn27A^1^*, *PPO2* ^Δ^ double mutants show a stronger melanization phenotype compared to *Spn27A^1^* single mutants with pupae turning black and adults presenting large black spots and wing defects (**Fig. 6**). Accordingly *Spn27A^1^*, *PPO2* ^Δ^ double mutants exhibit higher lethality than *Spn27A^1^* mutants (**Fig. S3**). These observations are consistent with higher PO activity in *PPO2* ^Δ^ and *Spn27A^1^* mutants compared to the wild-type.

### Melanization is dispensable for Toll and Imd pathway activation

**Figure 7:**
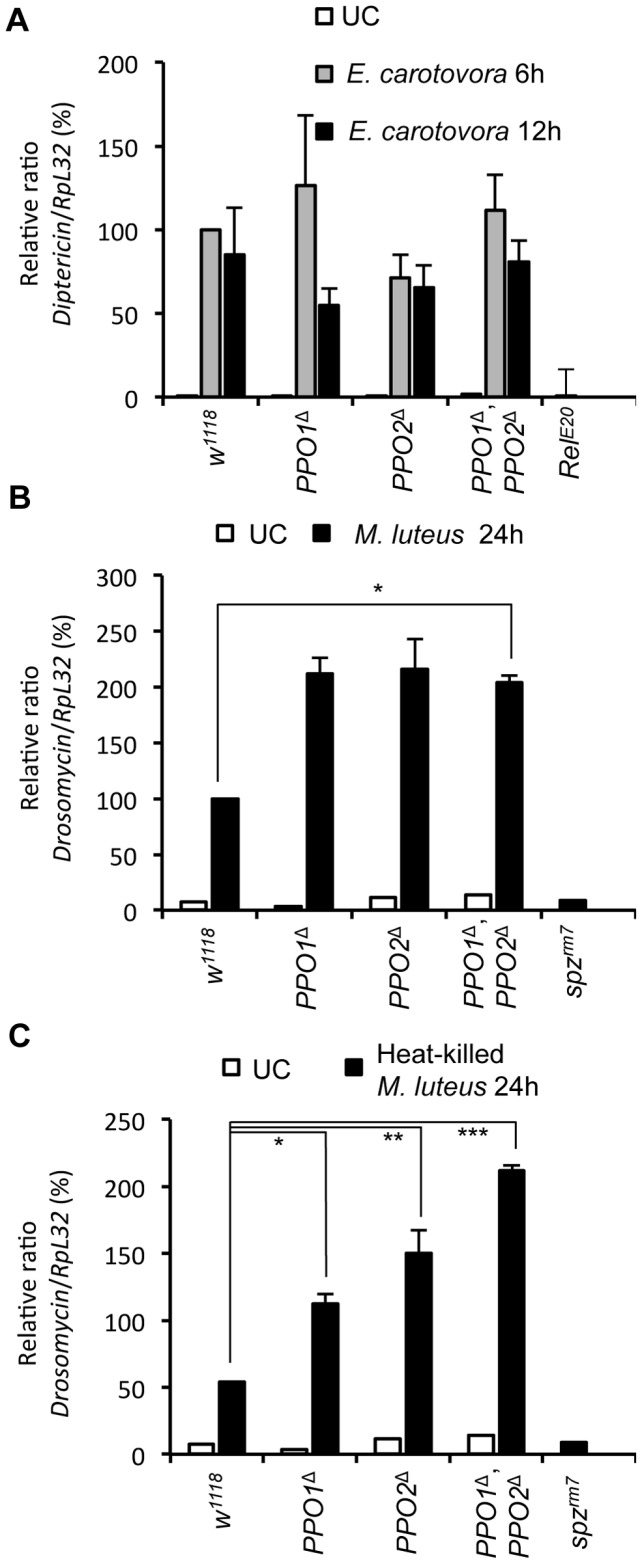
Melanization is not required for Toll and Imd pathway activities. (**A**) Expression of *Diptericin* in *PPO* mutant flies. Total RNA was extracted from animals either uninfected or collected 6 h and 12 h after septic injury with Gram-negative bacteria *E. carotovora*. Shown are the relative expression levels of *Dpt* in relation to *RpL32*. Single and double *PPO* mutants as well as *Bc* flies have wild-type *Dpt* expression levels. The Imd pathway mutant *Relish* was used as a negative control. (**B**) Expression of *Drosomycin* in *PPO* mutant flies 24 h after septic injury with Gram-positive bacteria *M. luteus* show that *PPO1* ^Δ^, *PPO2* ^Δ^ mutant flies (*,p<0.05) have an enhanced Toll pathway activity compared to wild-type. (**C**) *PPO1* ^Δ^ (*, p<0.05), *PPO2* ^Δ^ (**, p<0.005), and double *PPO1* ^Δ^, *PPO2* ^Δ^ mutant flies (***, p<0.0005) have an enhanced Toll pathway activity compared to wild- type flies upon infection to heat-inactivated *M. luteus*. Toll pathway mutant *spätzle^rm7^* was used as a negative control. Shown are the relative expression levels of *Drs* in relation to *RpL32*. 100% corresponds to *Drs* expression level of wild-types flies 24 h after septic injury with the Gram-positive bacteria *M. luteus*. Data were analyzed using one-way ANOVA and post-test and values represent the mean±s.e. of at least three independent experiments.

Septic injury induces the production of antimicrobial peptides by the fat body [35]. This reaction is regulated at transcriptional level by the Toll and the Imd pathways. Previous studies suggest that antimicrobial peptide gene expression is not affected by melanization [13], [18], [36]. To extend this analysis, we measured the expression of Toll and Imd target genes in single and double *PPO* mutant adults. We found that *PPO* mutations have no significant effect on the expression of *Diptericin*, an antibacterial peptide gene tightly regulated by the Imd pathway, in both unchallenged and *Erwinia*-infected conditions (**Fig. 7A**). However, the antifungal peptide gene *Drosomycin*, a target of the Toll pathway, is induced at higher levels in *PPO* mutants upon challenge with the Gram-positive *M. luteus* bacterium (**Fig. 7B**). To test whether this effect is caused by an increased proliferation of *M. luteus* in *PPO* mutants, we repeated this experiment using heat-killed bacteria. **Fig. 7C** shows that *Drosomycin* is still induced at significantly higher levels in *PPO* mutants, with a stronger effect in the double mutant than in single mutants. Since the expression of *Diptericin* and *Drosomycin* is controlled by the Imd and Toll pathways, respectively, we conclude that although *PPOs* are not required for Toll and Imd pathway activation, absence of melanization specifically enhances Toll activation.

### Melanization contributes to survival to wounding and to infection with Gram- positive bacteria

**Figure 8:**
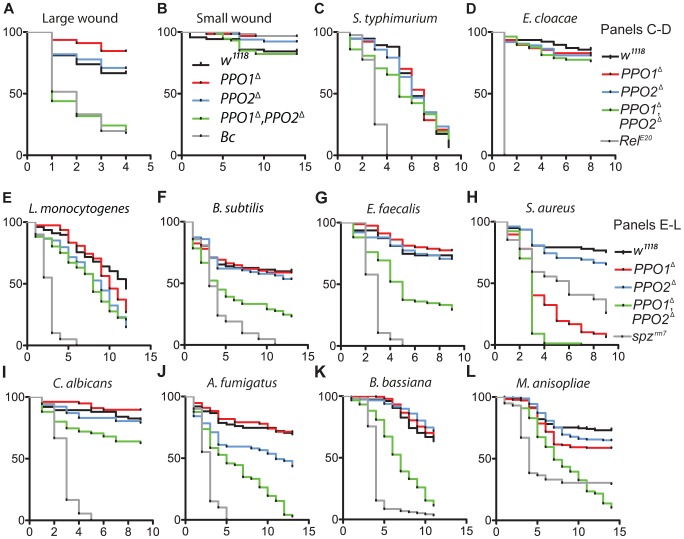
Contribution of *PPO1* and *PPO2* to wound healing and to host defense. (**A, B**) Survival rate of flies following injury with clean needle show that concomitant deletion of *PPO1* and *PPO2* impairs the capacity of flies to survive severe wounding (*p*<0.0001) compared to wild-type flies. (**C, D**) PPO1 and PPO2 are not required to survive septic injury with Gram-negative bacteria (*S. typhimurium* and *E. cloacae*). (**E, F**) *PPO1* ^Δ^, *PPO2* ^Δ^ mutant flies are less resistant to infections with Gram-positive DAP-type bacteria *L. monocytogenes (p* = 0.0002) and *B. subtilis (p*<0.0001) compared to wild-type animals. (G, H) PPO1^Δ^, *PPO2* ^Δ^ mutant flies are severely affected in their capacity to resist infection with Gram-positive Lys-type bacteria *E. faecalis* and *S. aureus (p*<0.0001). *PPO1* ^Δ^ and *PPO1* ^Δ^, *PPO2* ^Δ^ flies are even more susceptible to *S. aureus* than *spz^rm7^ flies p*<0.0001. *PPO1* ^Δ^ mutant flies also exhibit a reduced survival to *S. aureus* infection (*p*<0.0001). (**I, J**) *PPO1* ^Δ^, *PPO2* ^Δ^ mutant flies exhibit a moderate susceptibility upon septic injury with the yeast *C. albicans (p* = 0.0101) and a strong susceptibility to injection of *A. fumigatus* spores *(p*<0.0001). *PPO2* ^Δ^ mutant flies also die significantly faster from *A. fumigatus* infection (*p* = 0.0018 (**K, L**) Natural infection with entomopathogenic fungi *B. bassiana* and *M. anisopliae* reveals that *PPO1* ^Δ^, *PPO2* ^Δ^ flies have a reduced survival rate compared to wild-type flies (*p*<0.0001); so did *PPO1* ^Δ^ single mutant flies (*p* = 0.0084). Black cells (A, B), *Relish^ E20^* (C, D) and *spätzle^rm7^* (E, L), respectively, were used as controls. x-axis: Time post-infection in days; y-axis: Percentage of living flies. Data were analyzed by Log-rank test and values are pooled from three independent experiments. Survival to *E. faecalis*, *S. aureus* and *B. bassiana* have been repeated using fly lines carrying the *PPO* mutations in an OregonR background (**Fig. S4C–E**).

The *in vivo* importance of melanization in wound healing and host defense has so far been investigated by perturbing the function of proteases required to activate PPOs or by using the uncharacterized *Black cells* (*Bc*) mutation [17], [18], [26], [27], [36], [37]. Our *PPO1* ^Δ^ and *PPO2* ^Δ^ mutants now provide ideal tools to precisely assess the role of PPOs in survival to infection, as well as the individual contribution of PPO1 and PPO2 to host defense. To test the role of PPOs in wound healing, we monitored the survival of flies after mild or severe wounding. This was achieved by perforation of the thorax with clean needles of two different diameters as described in Nam *et al.* [13]. No significant difference was observed between mutants and wild-type upon mild injury. However, *PPO1* ^Δ^, *PPO2* ^Δ^ flies but not the single mutants exhibit a higher susceptibility to severe injury compared to wild-type (**Fig. 8A**–**B**). This confirms a role of PPOs in wound healing [13], [27] and shows that both PPO1 and PPO2 contribute to the recovery after injury.

**Figure 9:**
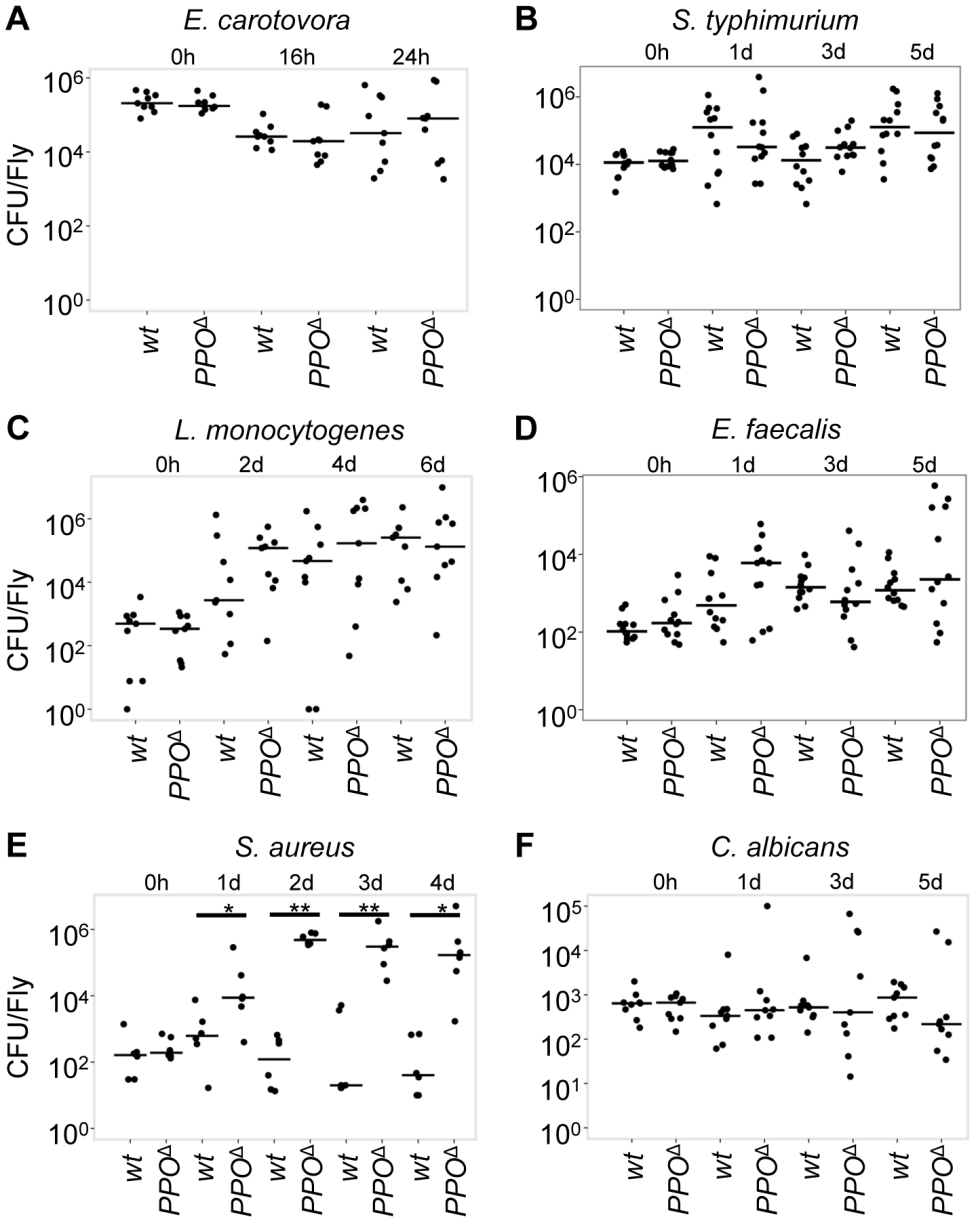
Microbial persistence in wild-type and *PPO1* ^Δ^, *PPO2* ^Δ^ double mutant flies. Persistence of *E. carotovora* (**A**), *S. typhimurium* (**B**), *L. monocytogenesis* (**C**), *E.* *faecalis* (**D**), *S. aureus* (**E**) and *C. albicans* (**F**) were evaluated at different times post- infection in wild-type and *PPO1* ^Δ^, *PPO2* ^Δ^ mutant flies (referred to as *PPO* ^Δ^). Statistical analysis reveals no bacterial persistence difference in *PPO1* ^Δ^, *PPO2* ^Δ^ double mutants compared to wild-type flies upon *E. carotovora, S. typhimurium, L. monocytogenesis, E. faecalis* and *C. albicans* infections. However, we noticed an increased counts of *S. aureus* in *PPO1* ^Δ^, *PPO2* ^Δ^ double mutants compared to wild-type flies (1 day: *p* = 0.04113; 2 days: *p* = 0.002165; 3 days: *p* = 0.004922; 4 days: *p* = 0.009524). Bars  =  median. The number of colony forming units (CFU) is expressed per fly and in a logarithmic scale. Data were analyzed by Wilcoxon test and values are pooled from three independent experiments.

We next used *PPO* mutant flies to ask to what extent melanization is important for survival to microbial infection, as this question has long been debated [17], [18], [26]. Flies were infected with various Gram-negative and Gram-positive bacteria. The survival rates of *PPO* flies were similar to wild-type flies after infection with *Salmonella typhimurium* and *Enterobacter cloacae* (**Fig. 8C, D**) while the *PPO1* ^Δ^, *PPO2* ^Δ^ flies were more susceptible to *Erwinia carotovora* (**Fig. S4A**). We next investigated the role of PPOs in the defense against Gram-positive bacteria and compared it to that of the *spz^rm7^* Toll pathway mutant. *L. monocytogenes* and *B. subtilis* are two Gram-positive bacteria with diaminopimelic (DAP)-type peptidoglycan that can activate both Toll and Imd pathways [38]. **Fig. 8E, F** shows that *PPO1* ^Δ^, *PPO2* ^Δ^ double mutants have an intermediate susceptibility between wild-type and *spz^rm7^* upon infection with these bacteria. Interestingly, *PPO* flies were also significantly susceptible to infection with Gram-positive bacteria with Lysine-type peptidoglycan (*E. faecalis*, *S. aureus, S. saprophyticus*), which are known to activate the Toll pathway exclusively (**Fig. 8G, H** **and S4B–D**). Surprisingly, both *PPO1* ^Δ^ and *PPO1* ^Δ^, *PPO2* ^Δ^ double mutant flies show a higher mortality compared to *spz^rm7^* upon *S. aureus* infection. Accordingly, the increased susceptibility of *PPO* mutants to *S. aureu*s was associated with a higher bacterial load indicating that in this specific case, PPOs contribute to pathogen containment (**Fig. 9E**). In contrast, the persistence of the Gram- negative bacteria *E. carotovora* and *S. typhimurium* and the Gram-positive bacteria *L. monocytogenesis* and *E. faecalis* was not significantly affected in the *PPO1* ^Δ^, *PPO2* ^Δ^ double mutants (**Fig. 9A**–**D**).

Taken together, the survival phenotypes of *PPO* mutants indicate that PPOs cooperate with the Toll pathway in promoting host defense against Gram-positive bacteria, notably Lysine-type strains.

### PPOs contribute to survival to infection with entomopathogenic fungi

We finally investigated the role of melanization in the survival to fungal infections using two infection protocols. In the first, yeast (*C. albicans*) or spores of a filamentous fungus (*A. fumigatus*) were injected into the body cavity. In the second, we mimicked natural infection by covering flies with spores of entomopathogenic fungi (*B. bassiana*, *M. anisopliae*). Hyphae from germinated spores can penetrate the cuticle and grow within the body cavity, eventually killing flies within several days [39]. *PPO* mutants exhibit significantly higher susceptibility to injected *C. albicans* compared to wild-type flies although no effect could be detected on the persistence of this yeast (**Fig. 8I** **and** **Fig. 9F**). This would suggest a role of PPO in the tolerance to *C. albicans* (*i.e.* an increased capacity to endure the consequences of infection). More strikingly, injection of spores of *A. fumigatus* or natural infection with *B. bassiana* and *M. anisopliae* lead to a marked vulnerability of *PPO1* ^Δ^, *PPO2* ^Δ^ double mutants compared to wild-type animals (**Fig. 8J**–**L, S** **4E**).

Collectively, our study demonstrates that melanization significantly contributes to the survival to fungal and Gram-positive bacterial infections. Higher susceptibilities to infection are observed in *PPO1* ^Δ^, *PPO2* ^Δ^ double mutants and sometimes in *PPO1* ^Δ^ single mutants, consistent with the lower levels of PO activity in these flies.

## Discussion

Although melanization is a well-established immune reaction of insects, the precise role of PO genes has never been addressed by loss-of-function mutations. In this study, we have generated deletions of *PPO1* and *PPO2*. We observed that *PPO* mutants are viable and do not show any pigmentation defect. This is in agreement with the early assumption that another type of enzymes, laccases, but not POs, participate in cuticle tanning [40]. An important finding is that *PPO1* ^Δ^, *PPO2* ^Δ^ double mutants do not develop any hemolymphatic PO activity upon wounding or following microbial infection. This indicates that PPO1 and PPO2 are the two main sources of PO activity in the hemolymph of *Drosophila*. Antimicrobial peptide genes such as *Diptericin* and *Drosomycin* remain inducible in *PPO1* ^Δ^, *PPO2* ^Δ^ double mutants. This is consistent with many studies, which showed that PO activity is not required for Toll and Imd pathway activation [13], [18], [36]. Interestingly, Toll pathway activation tends to be stronger in *PPO* deficient flies, as revealed by a higher level of *Drosomycin* expression. Higher Toll activity was also observed upon injection of dead bacteria, indicating that this effect is not caused by an increased bacterial proliferation in melanization deficient flies. A possible explanation is that melanin alters the microbes rendering their PAMPs (most probably peptidoglycans) less accessible to pattern-recognition receptors that act upstream of the Toll pathway. Since the PPO and Toll cascades are downstream of the same Pattern-Recognition Receptors [25], another explanation is that loss of one downstream arm frees up effector proteases for the other arm, resulting in over-activation of Toll signaling in *PPO* mutants.

The contribution of melanization in *Drosophila* host survival to either injury or microbial infection has been addressed using various mutations: *Black cells* [17], [27], [36], [37], *Sp7* [17], [18], [26] and *PPO1* ^Δ^, *PPO2* ^Δ^ (this study) (summarized in **Table 1**). Collectively, Table 1 shows that *PPO1* ^Δ^, *PPO2* ^Δ^ double mutants, *Black cells*, and *Hayan* ^1^ mutants behave the same way in agreement with the fact that they all lack hemolymphatic PO activity. *PPO1* ^Δ^, *PPO2* ^Δ^, *Black cells*, and *Hayan* ^1^ flies are viable but exhibit increased susceptibility to large injury ([11], [27], this study) and to Gram-positive bacteria ([17], this study) and fungi ([14], [17], this study). Based on these observations, we can conclude that melanization plays a non-redundant role in the defense against Gram-positive bacteria and fungi. This is also supported by studies using the *Sp7* mutants [26]. The contribution of PO to survival to Gram-negative infection is less clear as *PPO1* ^Δ^, *PPO2* ^Δ^ flies survived like wild-type to *E. cloacae* infection while exhibiting a mild susceptibility to *E. carotovora* (this study). Nevertheless, *Black cells* flies have been reported to be more susceptible to *A. tumefaciens* [17]. Contradictory results have been observed for the survival to *S. typhimurium* ([26], this study) that could be explained by the strain used. However, previous experiments have shown that flies lacking both a functional Imd pathway and the *Black cells* mutation die faster to infection with *E. coli* [18], [36]. Collectively, this suggests that PO contributes to the survival to Gram-negative infection but its effect is either modest or redundant with other mechanisms.

**Table 1:** Contribution of melanization to survival and microbial infection.

		Genotype							
Challenge		Sp7/MP2^Δ^		*MP1-RNAi*	*Sp7-RNAi*	*Hayan* ^1^	*Black cells*	*PPO1* ^Δ^, *PPO2* ^Δ^	
		Survival	Microbe load	Survival	Survival	Survival	Survival	Survival	Microbe load
Wounding	Small	Lower *^ 5^						Same ^7^	
	Large			Same ^6^	Same ^6^	Lower * ^6^	Lower * ^1, 6^	Lower * ^7^	
Gram-negative bacteria	*E. coli*	Same ^4, 5^	Same ^5^						
	*E. carotovora*			Same ^3^	Same ^3^			Lower * ^7^	Same ^7^
	*S. typhimurium*	Lower * ^5^	Higher * ^5^					Same ^7^	Same ^7^
	*E. cloacae*							Same ^7^	
	*B. cepacia*	Same ^5^	Higher * ^5^						
	*A. tumefaciens*	Same ^4^					Lower ^4^		
Gram-positive (Lys-type PGN) bacteria	*E. faecalis*	Same ^4, 5^	Lower * ^5^	Same ^3^	Same ^3^		Lower ^4^	Lower * ^7^	Same ^7^
	*S. aureus*	Lower * ^5^ + Same ^4^						Lower * ^7^	Higher * ^7^
	*S. saprophyticus*							Lower * ^7^	
	*S. pneumoniae*	Higher * ^5^	Lower * ^5^						
Gram-positive (DAP-type PGN) bacteria	*L. monocytogenes*	Lower * ^5^	Higher * ^5^					Lower * ^7^	Same ^7^
	*B. subtilis*							Lower * ^7^	
Yeast	*C. albicans*			Same ^3^	Same ^3^			Lower * ^7^	Same ^7^
Entomopathogenic fungi	*B. bassiana*	Same ^4^		Same ^3^	Lower ^3^		Lower^ 2,^ ^4^	Lower * ^7^	
	*M. anisopliae*							Lower * ^7^	
	*A. fumigatus*							Lower * ^7^	

This table summarizes several survival and bacterial load analyses performed with mutations affecting the melanization cascade in *Drosophila*. Lower, Higher and Same mean lower, higher or same host survival (or bacterial load) compared to wild-type, respectively. The asterisk (*) symbol indicates a statistical p value <0.05. The references are given as superscript numbers: 1. [27]; 2. [14]; 3. [18]; 4. [17]; 5. [26]; 6. [13]; 7. this study.

Our persistence analysis did not detect any significant difference in bacterial loads in wild-type and *PPO1* ^Δ^, *PPO2* ^Δ^ flies upon infection with *E. carotovora*, *L. monocytogenes*, *E. faecalis* and *C. albicans*, despite an increased susceptibility to these microbes. A first explanation is that PPOs do contribute to tolerance rather than resistance to these micro- organisms [41]. Nevertheless, we cannot exclude that a role in resistance remained undetectable due to the high variability of bacterial load measurements. We point out that work by others [26], which used injection techniques producing less variance demonstrated a role for melanization in the control of *L. monocytogenes* and *S. typhimurium*. In contrast, a higher number of bacteria were found in *PPO* ^Δ^ flies infected with *S. aureus*. Since *PPO* mutations do not affect the systemic antimicrobial response, this indicates that PO is an immune effector mechanism involved in the resistance to *S. aureus*. This is in line with several studies that have shown direct microbicidal activity of PO [1], [42], [43]. Except for *S. aureus* infection, *PPO1* ^Δ^, *PPO2* ^Δ^ flies tend to cope better with infection than *spz^rm7^* flies, which lack a functional Toll pathway. Since the Toll pathway contributes to PO activation in adults [15], [44], our survival analysis suggests that a significant fraction of Toll-mediated killing is likely caused by PO activity. There are several explanations why *PPO* mutant flies die faster than Toll pathway mutants upon *S. aureus* infection. PO could be the only defense mechanism against this bacterium, and the residual PO activity in Toll deficient flies could contribute to survival. A Toll- independent immediate melanization reaction might contain the pathogen and prevent its dissemination at the early steps of infection. Alternatively, the Toll immune response could comprise factors that are deleterious for the host in the absence of melanization. Since phagocytosis also plays an important role in the survival to *S. aureus* [45]–[47], future studies should investigate the precise link between PPO, phagocytosis and Toll pathway activation during *S. aureus* infection. Beyond PO's own microbicidal activity, we cannot exclude additional roles for this enzyme, such as for example protection of the host from immunopathology by sequestering microbicidal activities in the immediate proximity of the pathogen. The important role of melanization in the fight against entomopathogenic fungi is consistent with other reports [48] and with the observation that many entomopathogenic fungi have evolved anti-PO activity, as illustrated namely by an active PO suppression by *B. bassiana* [25].

The number of *PPO* genes varies among insects from one in the honeybee to ten in *Aedes aegypti* [49]. This variation in gene number probably reflects differences in the importance of this immune reaction in different insect species. Higher numbers of *PPO* genes could increase the flexibility in their use allowing complex spatio-temporal deployments of POs at the various insect life stages. *Drosophila* has three PPOs and our study points to overlapping and distinct functions for all three. Although PPO1 and PPO2 both contribute to hemolymph PO activity, these PPOs are not fully redundant. Our study suggests that PPO1 is involved in the rapid delivery of PO activity when required, while PPO2 provides a storage form that can be deployed in a second phase. This is supported by the observation that melanization at the wound site is delayed in *PPO1* ^Δ^ larvae and adults. Moreover, our data show that PPO2 contributes to the crystals in crystal cells as none were found in *PPO2* ^Δ^ mutants. We hypothesize that PPO1 is also produced in crystal cells but rather than stored is directly secreted into the hemolymph. In our study we observed a precocious activation of PPO1 in the absence of PPO2 pointing to an interaction between both PPOs, most probably within the crystal cells. Crystals would provide an appropriate means of bulk storage and rapid release of concentrated PPO upon crystal cell rupture. Since crystal cells are found in larvae, this might reflect a predominant role of melanization at this stage. Indeed, melanization is thought to play an important role in the encapsulation of parasitoid wasp eggs, which specifically target larval stages. Thus, crystal cells could have evolved as an adaption to release a large quantity of PO in larvae while the rapid delivery of PPO may be less relevant in adults.

Western blot analysis shows an increase of PPO1 and its mature form PO1 in *PPO2* ^Δ^ mutants compared to the wild-type. Since both PPO1 and PPO2 contribute to hemolymphatic PO, we conclude that PPO2 to some extent negatively impacts PPO1 activity. An interaction between PPO1 and PPO2 is suggested by the fact that *Black cells*, a mutation associated with PPO1, affects both PPO1 and PPO2 as it shows no hemolymph PO activity [5](unpublished data). This higher PO activity in the *PPO2* ^Δ^ mutant could be due to a problem of compartmentalization/secretion of the two enzymes in the crystal cell, a destabilization of PPO1 by PPO2 or to competition between PPO1 and PPO2 for cleavage by the same protease.

It is noteworthy that in both larvae and adults, transcription of *PPO* genes is not induced by infection or injury, while that of genes encoding other enzymes (*dopadecarboxylase*, *yellow*…) or serine proteases involved in the melanization reaction is [50]. This suggests that PO activity is mostly regulated at the post-transcriptional level. PPO3 is a lamellocyte specific PPO [10], [11] that does not require proteolytic cleavage for activation [12]. Our results show that PPO3 does not significantly contribute to hemolymph PO activity since no PO activity was observed in the absence of PPO1 and 2. In addition, we observed that capsules around parasitoid wasp eggs were not melanized in *PPO1* ^Δ^, *PPO2* ^Δ^ larvae, indicating that PPO3 by itself is not sufficient for this process to occur. Functional characterization of this PPO awaits the generation of a *PPO3* mutant. Complex regulatory cascades in the hemolymph converge on the terminal SP Hayan, which is thought to be directly involved in the cleavage of PPO1 and possibly PPO2 [13]. An open question remains whether PPO1 and PPO2 are differentially regulated by these cascades. Our epistatic analysis revealed that most of the *Spn27A^1^* phenotype is suppressed in the combined absence of PPO1 and 2. Previous studies have shown that mutations in the SP gene *Hayan* also rescue the *Spn27A^1^* phenotype [13]. This is consistent with the observation that Hayan and POs function downstream of Spn27A. We noticed that the removal of PPO1 or PPO2 differentially affects the *Spn27A^1^* phenotype. *PPO2*, *Spn27A^1^* flies exhibit higher levels of spontaneous PO activity compared to *Spn27A^1^* flies consistent with the notion that *PPO2* negatively impacts *PPO1*. Moreover, *PPO2* ^Δ^, *Spn27A^1^* and *PPO1* ^Δ^, *Spn27A^1^* larvae have a distinctive melanization pattern with hemolymphatic melanotic tumors in the former, and conspicuous dots in the latter. These two distinct patterns could arise from the different localization of PPO1 and PPO2 in the hemolymph and the crystal cells respectively, as suggested by our results.

Hemolymphatic melanotic tumors will be observed in *PPO2* ^Δ^, *Spn27A^1^* as Spn27A could inhibit PPO1 in the hemolymph compartment while melanization in dots could be observed in the *PPO1* ^Δ^, *Spn27A^1^* background *as* PPO2 activation could be inhibited by Spn27A inside the crystal cells residing in the hematopoietic sessile niche. The fact that the lethality observed in the absence of Spn27A is largely rescued in the absence of both PPO1 and PPO2 suggests that a significant part of this lethality is caused by the constitutive activation of PO in these mutants. Nevertheless, the residual lethality in *Spn27A^1^* flies devoid of *PPO1* and *PPO2* indicates the existence of lethality independent of PO activity itself. This could be due to other functions or to damage caused by the presence of activated SPs in the hemolymph.

As a final note, our mechanistic understanding of *Drosophila* immune effector mechanisms is still very limited. In-depth mechanistic analysis is hampered by the fact that many immune effector genes (such as *TEPs*, *antimicrobial peptide* genes, *POs*) are present as several members and likely function redundantly. This is actually illustrated by the present study since high susceptibility to infection was observed only when two PPOs were knocked-out simultaneously. Fortunately, the development of new knock-out strategies [51], [52], combined with genetics, offers the possibility to dissect the function of each member of a gene family as well as the family's overall contribution. From this point of view, our study definitively confirms the important role of phenoloxidases in insect host defense.

## Materials and Methods

### Insects stocks and mutant generation

Unless indicated otherwise, *w^1118^* or OregonR flies were used as wild-type controls. The *Relish^E20^* (*Rel^E20^*), *spätzle^rm7^* (*spz^rm7^*), *Black cells* (*Bc*) *Serpin27A^1^* (*Spn27A^1^*) and *UAS-bax/CyO-actin-GFP* lines are described in [14], [36], [53], [54]. The *Lz*-*Gal4*, *UAS*-*GFP* line was obtained from the Bloomington Stock Center. The parasitoid wasp *Asobara tabida* (Hymenoptera: Braconidae), was reared on *PPO1* ^Δ^, *PPO2* ^Δ^ double mutant fly stocks at room temperature. After emergence wasps were kept at 16°C and provided with honey until use for experimentation. The *PPO1* ^Δ^ KO line was generated by homologous recombination (**Fig.1A**) [55]. 4 kb and 4.3 kb of DNA sequence flanking the 5′ and 3′ ends, respectively, of the *PPO1* locus (**Fig. 1A**) were cloned into the p{W25} vector [55]. Flies transformed with p{W25} were used to generate a deletion of *PPO1* using a standard crossing protocol. We confirmed by PCR that *PPO1* was replaced by the *w+* gene in *PPO1* ^Δ^ mutants using forward primer 5′- CCATCTCCAGAATGCCCCCTTCACT-3′ and reverse primer 5′- TGCTCAACGGAGTCCTGCGAGTAAT-3′. The *PPO2* ^Δ^ KO line was generated by mobilization of the transposable element *Mi{ET1}proPO45^ MB05593^* inserted in the 3′ end of *PPO2* following standard P-element excision protocol. The imprecise excision deleted a fragment of 5.2 kb including the non-coding sequence of the neighboring gene *CG13743* without affecting its expression (see **Fig.1A** and **Fig. S1**). Primers used to determine the size of deleted fragments can be obtained on request. *PPO1* ^Δ^, *PPO2* ^Δ^ double mutant flies were generated by standard crosses. Both *PPO* deficient lines were initially backcrossed five times into a *w^1118^* or OregonR wild-type strain to homogenize the background. *Drosophila* stocks were maintained at 25°C on standard fly medium. Germ-free animals were generated as described previously [56].

### Microorganism culture and infection experiments

The bacterial strains used and their respective optical density of the pellet (O.D.) at 600 nm were: the Gram-negative bacteria *Erwinia carotovora 15*-*GFP* (*E. carotovora*, O.D. 200), *Enterobacter cloacae β12* (*E. cloacae*, O.D. 10) and *Salmonella typhimurium pFVP 25.1* (*GFP*) (*S. typhimurium*, O.D. 10); the DAP-type PGN containing Gram-positive bacteria *Listeria monocytogenes-GFP* (*BUG2377*) (*L. monocytogenes*, O.D. 0.5) and *Bacillus subtilis* (*B. subtilis*, O.D 5); the Lys-type PGN containing Gram-positive bacteria *Micrococcus luteus* (*M. luteus*, O.D. 200), *Staphylococcus aureus-GFP* (*S. aureus*, O.D.0.5), *Staphylococcus saprophyticus* (*S. saprophyticus*, O.D. 5) and *Enterococcus faecalis* OG1RF+pMV158GFP [57] (*E. faecalis*, O.D. 0.5); the yeast *Candida albicans* (*C. albicans,* O. D. 200). Strains were cultured in Brain-Heart Infusion Broth (BHI - *L. monocytogenes*), Yeast extract-Peptone-Glucose Broth (*C. albicans*) or Luria Broth (LB - all others) at 29°C (*E. carotovora*, *M. luteus, C. albicans*) or 37°C. Spores of entomopathogenic strains *Beauveria bassiana 802* (*B. bassiana*) and *Metarhizium anisopliae* KVL 131 (*M.anisopliae*) were grown on Malt agar plates at 29°C for approximately 3 weeks until sporulation. Systemic infections (septic injury) were performed by pricking adult females in the thorax with a thin needle previously dipped into a concentrated pellet of a bacterial culture or in a suspension of *Aspergillus fumigatus* (*A. fumigatus*) spores [58]. Natural infections were initiated by shaking anesthetized flies in a petri dish containing a sporulating culture of entomopathogenic fungi *B. bassiana* or *M. anisopliae*. Infected flies were subsequently maintained at 29°C (*E. carotovora*, *M. luteus, B. bassiana*, *M. anisopliae* and *A. fumigatus*) or at 22°C (all other bacteria) [39]. At least two tubes of 20 flies were used for survival experiments and survival was scored daily. Experiments were repeated at least three times. For lifespan experiments, flies were kept on normal fly medium or on sterilized medium for axenic flies and were flipped every three days. Persistence assay: Flies were infected with the pathogens as described above. Persistence was evaluated at different time points post-infection in triplicates by crushing 5 flies in LB culture medium. Serial dilutions of these extracts were spotted in triplicate on appropriate medium and incubated overnight at 29°C or 37°C. Colonies were counted from spots containing more than 10 colonies. The experiment was repeated three times. For wasp infection, 20 first instar wild-type or *PPO* larvae were placed in presence of 3 *A.tabida* females for 2 hours. Parasitized larvae were kept at room temperature until capsule dissection 6 days post-infection. We noticed that the wasp larvae develop better in the *PPO1* ^Δ^, *PPO2* ^Δ^ mutants compared to the wild-type as *A. tabida* could be more easily maintained in *PPO1* ^Δ^, *PPO2* ^Δ^ than in the *w* *^1118^* or OregonR wild-type stocks.

### Wounding experiment

‘Clean’ injury referred to an injury performed with a needle that has been previously sterilized. A low level of bacterial contamination is still possible since the surface of the insect was not sterilized. For adults, two different intensities of physical wounding were used (adapted from [13]). In strong wounding the thorax of the fly was completely penetrated using a sterile needle of ∼50 µm diameter. For survival to clean injury and for imaging of the melanization reaction upon pricking, the thorax of the animal was pricked (as described in infection experiments) using a sterile needle (diameter: ∼5 µm). Pictures were taken three hours post-pricking. Third instar larvae were pricked dorsally using a sterile needle (diameter: ∼5 µm). Pictures of melanized larvae were taken 30 minutes post-injury. For the melanization time-course of larvae (**Fig. 3B**), the presence of a blackening spot was recorded after pricking, every 5 minutes until 30 minutes. Pictures were captured with a Leica DFC300FX camera and Leica Application Suite.

### Hemolymph extraction and PO activity

Larval and adult hemolymph was collected as follows. Fifteen to twenty individuals were placed on a 10 µM filter of an empty mobicol spin column (MOBITEC), covered with glass beads and centrifuged for 20 minutes at 4°C, 10'000 r.pm. Hemolymph was recovered in 50 µl protease inhibitor solution (Roche; one tablet dissolved in 4 ml phosphate-buffered saline, PBS) and protein concentrations adjusted after a Bradford test. Sample volumes were adjusted to 200 µl in 5 mM CaCl_2_ solution (diluted in protease inhibitor solution, see above) and after addition of 800 µl of L-DOPA solution (20 mM in phosphate buffer pH 6.6) the samples were incubated at 29 °C in the dark. After 30 min, the optical density at 492 nm was measured for each sample against an L-DOPA control containing no hemolymph. Since activation of the proPO system was blocked by the presence of the protease inhibitor, the values reflect the in vivo PO activity at the time of wounding. Each experiment was repeated three times.

### Western blots

For Western blots, hemolymph samples were collected from 25 flies in a protease inhibitor solution as described above. Protein concentration of the samples was determined by Bradford assay. 25 µg of protein extract was separated on a 4–20% acrylamide precast Novex gel (Invitrogen) under reducing conditions and transferred to nitrocellulose membranes (Invitrogen iBlot). After blocking in 2% bovine serum albumin in PBT for 1 h, samples were incubated at 4°C overnight with rabbit antibodies against *Drosophila* PPO1 or PPO2 [13] in a 1∶2000 dilution. Goat anti-rabbit-HRP secondary antibody (Dako) in a 1∶2000 dilution was incubated for 2 h at room temperature. Bound antibody was detected using ECL (GE Healthcare) according to the manufacturer's instructions.

### Live imaging and immunofluorescence

For crystal cells imaging, the hemolymph of wild-type or PPO mutant larvae expressing *Lz*-*Gal4*, *UAS-GFP* was immediately fixed on Superfrost Plus adhesive microscope slides (Menzel) with PBS:paraformaldehyde 4% and rinsed with PBS. *Lz-Gal4*, *UAS-GFP* expressing cells were stained with rabbit anti-PPO1 (1∶500) or rabbit anti-PPO2 (1∶500) [13] and with mouse anti-GFP (Interchim) diluted in PBT+1% BSA. Anti-PPO1and anti-PPO2 were revealed with an Alexa 594-coupled mouse anti-rabbit (Molecular Probes) diluted to 1∶500 in blocking solution (PBS, 0.1% Tween, 2% BSA). Anti-GFP was revealed with an Alexa 488-coupled mouse anti-mouse (Molecular Probes) diluted to 1∶500 in blocking solution and nuclei were stained with DAPI (Sigma).

For staining of capsules from *A. tabida* infected larvae, capsules were dissected from 6 day old infected larvae, fixed with PBS:paraformaldehyde 4% and rinsed with PBS. Lamellocytes on capsules were stained with AF594-conjugated Phalloidin (Molecular Probes) diluted 1∶40 in blocking solution. Samples were observed for fluorescence with an Axioplot imager Z1 and Axiocam mRM camera (Zeiss) and a 100/1.30 NA oil immersion objective, with zoom set to 100 (crystal cells) or 20 (capsules). For publication purposes, brightness and contrast were increased identically on control and sample images.

### Quantitative RT-PCR

For quantification of mRNA, whole flies were collected at indicated time points. Total fly RNA was isolated from 15 adult flies by TRIzol reagent and dissolved in RNase- free water. Five hundred nanogram total RNA was then reverse-transcribed in 10 µl reaction volume using PrimeScript RT (TAKARA) and random hexamer primers. Quantitative PCR was performed on a LightCycler 480 (Roche) in 96-well plates using the LightCycler 480 SYBR Green I master mix or on a LightCycler 2.0 (Roche) in capillaries using dsDNA dye SYBR Green I (Roche). Primers were as follows: *Diptericin* forward 5′- GCTGCGCAATCGCTTCTACT-3′, reverse 5′-TGGTGGAGTGGGCTTCATG-3′; *Drosomycin* forward 5′-CGTGAGAACCTTTTCCAATATGAT-3′, reverse 5′- TCCCAGGACCACCAGCAT-3′; *RpL32* forward 5′-GACGCTTCAAGGGACAGTATCTG-3′, reverse 5′-AAACGCGGTTCTGCATGAG-3′; *PPO1* forward 5′- TTTTCCCTACTGACAACCTC-3′, reverse 5′- GGGCTGATAGTCTGCTC-3′; *PPO2* forward 5′- CCCGCCTATACCGAGA-3′, reverse 5′- CGCACGTAGCCGAAAC-3′; *PPO3* forward 5′-GGCGAGCTGTTCTACT-3′, reverse 5′- GAGGATACGCCCTACTG-3′; *CG13743* forward 5′- GAAGGGCGTAGGGTAT-3′, reverse 5′- CCATCAGACACATCAGC-3′.

### Statistical analysis

Each experiment was repeated independently a minimum of three times (unless otherwise indicated), error bars represent the standard error of the mean of replicate experiments (unless otherwise indicated). Statistical significance of survival and persistence data was calculated with a log-rank or a Wilcoxon test, respectively, and p values are indicated in figure legends. Otherwise statistical significance was calculated with Student's t test or one-way ANOVA followed by post-test and p values of <0.05 =  *, <0.005 =  **, and <0.0005 =  *** were considered significant.

## Supporting Information

Figure S1 **PPO1 and PPO2 deletions only marginally affect the expression of other PPO genes.** (**A**) Expression of *PPO1* (left panel), *PPO2* (center panel) or *PPO3* (right panel) in *PPO* mutant flies upon a 24 h systemic infection with Gram-positive bacteria *M. luteus*. Expression of *PPO1* and *PPO2* is strongly reduced in the respective *PPO1^Δ^* and *PPO2^Δ^* mutants compared to wild-type. The expression of *PPO3* was slightly up-regulated in *PPO2^Δ^* flies. For (A) and (B) values represent the relative expression levels in relation to *RpL32*. Data were analyzed using t.test and values represent the mean±s.e. of at least three independent experiments. (**B**) The expression level of *CG13743* is not affected by the imprecise excision of *Mi{ET1}proPO45^MB05593^* inserted in the 3′ end of *PPO2* compared to wild-type flies under unchallenged conditions. (**C**) Both *PPO1* ^Δ^ and *PPO2* ^Δ^ single mutants and the *PPO1* ^Δ^, *PPO2* ^Δ^ double mutant were viable and did not exhibit any overt developmental or pigmentation defects. (**D**) *PPO1* ^Δ^, *PPO2* ^Δ^ double mutant flies exhibit faster death rates than wild-type (P = 0.0012) even when raised under axenic conditions. Each survival curve corresponds to one experiment of 3 vials of 20 flies each. p values were calculated using the Log-rank test.
(PDF)

Figure S2 **Melanization at the wounding site in PPO1 and PPO2 adults.** (**A**) Melanization of flies after pricking is abolished in the absence of PPO1 and PPO2 while a reduced melanization spot is observed in *PPO1* ^Δ^ mutants (see magnifications). *Bc* flies are used as a control for the absence of melanization whereas *Spn27A^1^* flies display a more intense melanization upon pricking [5], [14]. Black arrows indicate the pricking site. Flies were wounded with a tungsten needle and blackening of the wound was recordedhours later. A representative picture is shown for each genotype. (**B**) Similar results were obtained when using *PPO* mutants backcrossed five times into OregonR background.
(TIFF)

Figure S3 **Survival of** ***Spn27A^1^, PPO*** **mutant flies.** The lethality induced by *Spn27A^1^* deficiency was largely rescued in the absence of PPO1 and PPO2 while it was enhanced in the absence of PPO2. Data were analyzed by t test and values represent the mean ± s.e. percentage of homozygotes hatching (maximum expected = 100%). The progeny from two crosses of 5 heterozygous females and 5 males were analyzed.
(PDF)

Figure S4 **Contribution of** ***PPO1*** **and** ***PPO2*** **to host defense.** (**A**) Compared to wild- type *Drosophila*, flies mutated for *PPO1* and *PPO2* have a reduced survival rate upon infection with Gram-negative bacteria *E. carotovora (p*<0.0001). (**B**) Compared to wild- type *Drosophila*, flies mutated for *PPO1* and *PPO2* have a reduced survival rate upon infection with Gram-positive Lys-type bacteria *S. saprophyticus (p*<0.0001). (**C-E**) Survival analyses were performed with *PPOs* fly lines that were backcrossed five times into OregonR. (**C, D**) Contrary to wild-type, *PPO1* ^Δ^, *PPO2* ^Δ^ mutant flies were severely affected in their capacity to resist infection with Gram-positive Lysine-type bacteria *E. faecalis (p*<0.0001) (**C**) and *S. aureus (p*<0.0001) (**D**). (**E**) Natural infection with entomopathogenic fungi *B. bassiana* reveals that *PPO1* ^Δ^, *PPO2* ^Δ^ flies have a reduced survival rate compared to wild-type flies *(p*<0.0001); Imd and Toll pathway deficient flies *Relish^E20^* and *spätzle^rm7^*, respectively, were used as controls. x-axis: Time post-infection in days; y-axis: Percentage of living flies. Data were analyzed using Log rank test and values are pooled data from three independent experiments.
(PDF)
