# Supplementary figures and images for: Prophenoloxidase Activation Is Required for Survival to Microbial Infections in Drosophila
Source: PLoS Pathog. 2014 May 1;10(5):e1004067. doi: 10.1371/journal.ppat.1004067 (PMC4006879; doi:10.1371/journal.ppat.1004067)

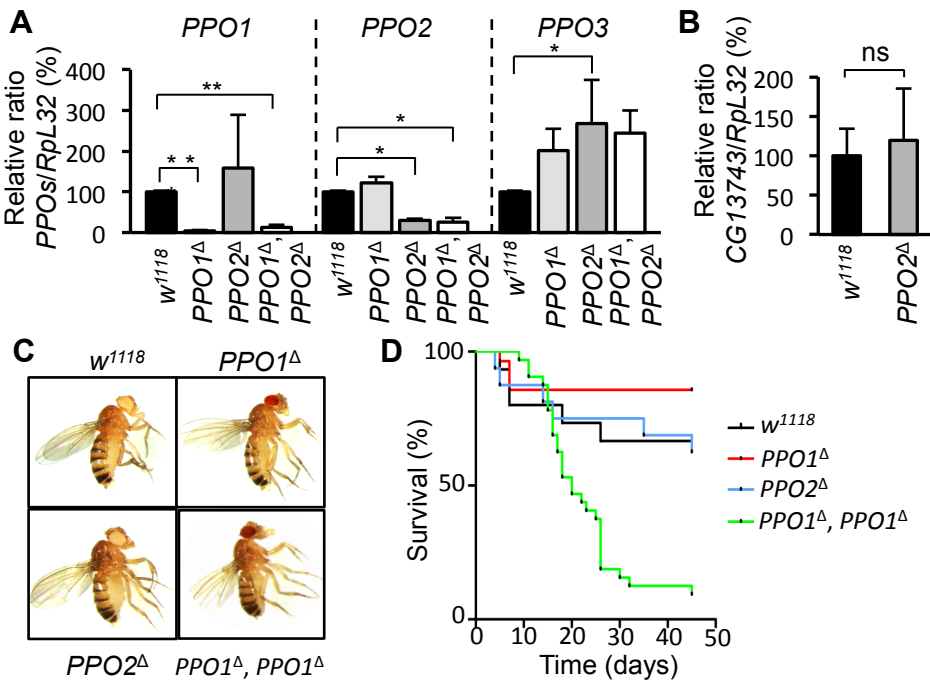

Supplement: Figure S1 — PPO1 and PPO2 deletions only marginally affect the expression of other PPO genes. (A) Expression of PPO1 (left panel), PPO2 (center panel) or PPO3 (right panel) in PPO mutant flies upon a 24 h systemic infection with Gram-positive bacteria M. luteus. Expression of PPO1 and PPO2 is strongly reduced in the respective PPO1Δ and PPO2Δ mutants compared to wild-type. The expression of PPO3 was slightly up-regulated in PPO2Δ flies. For (A) and (B) values represent the relative expression levels in relation to RpL32. Data were analyzed using t.test and values represent the mean±s.e. of at least three independent experiments. (B) The expression level of CG13743 is not affected by the imprecise excision of Mi{ET1}proPO45MB05593 inserted in the 3′ end of PPO2 compared to wild-type flies under unchallenged conditions. (C) Both PPO1 Δ and PPO2 Δ single mutants and the PPO1 Δ, PPO2 Δ double mutant were viable and did not exhibit any overt developmental or pigmentation defects. (D) PPO1 Δ, PPO2 Δ double mutant flies exhibit faster death rates than wild-type (P = 0.0012) even when raised under axenic conditions. Each survival curve corresponds to one experiment of 3 vials of 20 flies each. p values were calculated using the Log-rank test. (PDF) [file ppat.1004067.s001.pdf]

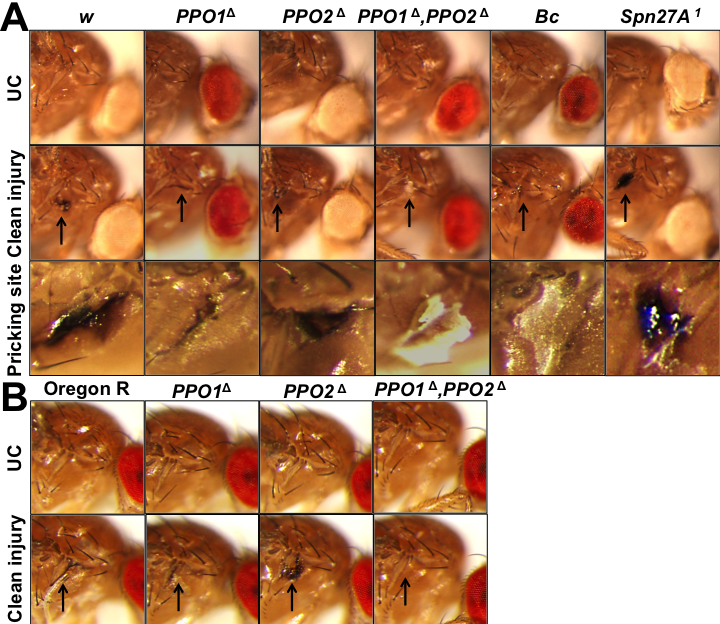

Supplement: Figure S2 — Melanization at the wounding site in PPO1 and PPO2 adults. (A) Melanization of flies after pricking is abolished in the absence of PPO1 and PPO2 while a reduced melanization spot is observed in PPO1 Δ mutants (see magnifications). Bc flies are used as a control for the absence of melanization whereas Spn27A1 flies display a more intense melanization upon pricking [5], [14]. Black arrows indicate the pricking site. Flies were wounded with a tungsten needle and blackening of the wound was recordedhours later. A representative picture is shown for each genotype. (B) Similar results were obtained when using PPO mutants backcrossed five times into OregonR background. (TIFF) [file ppat.1004067.s002.tif]

% expected homozygote flies  
surviving relative to balancer

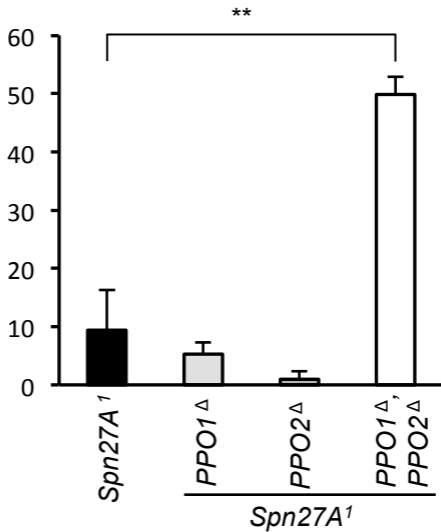

Supplement: Figure S3 — Survival of Spn27A1, PPO mutant flies. The lethality induced by Spn27A1 deficiency was largely rescued in the absence of PPO1 and PPO2 while it was enhanced in the absence of PPO2. Data were analyzed by t test and values represent the mean ± s.e. percentage of homozygotes hatching (maximum expected = 100%). The progeny from two crosses of 5 heterozygous females and 5 males were analyzed. (PDF) [file ppat.1004067.s003.pdf]

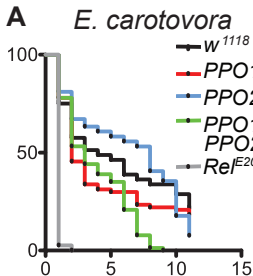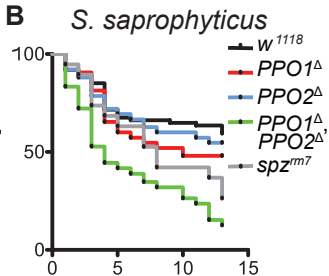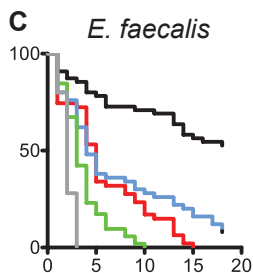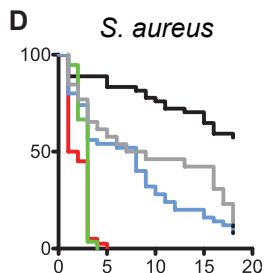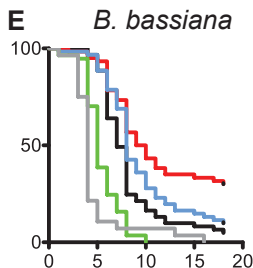

Panels C-E

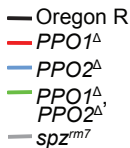

Supplement: Figure S4 — Contribution of PPO1 and PPO2 to host defense. (A) Compared to wild- type Drosophila, flies mutated for PPO1 and PPO2 have a reduced survival rate upon infection with Gram-negative bacteria E. carotovora (p<0.0001). (B) Compared to wild- type Drosophila, flies mutated for PPO1 and PPO2 have a reduced survival rate upon infection with Gram-positive Lys-type bacteria S. saprophyticus (p<0.0001). (C-E) Survival analyses were performed with PPOs fly lines that were backcrossed five times into OregonR. (C, D) Contrary to wild-type, PPO1 Δ, PPO2 Δ mutant flies were severely affected in their capacity to resist infection with Gram-positive Lysine-type bacteria E. faecalis (p<0.0001) (C) and S. aureus (p<0.0001) (D). (E) Natural infection with entomopathogenic fungi B. bassiana reveals that PPO1 Δ, PPO2 Δ flies have a reduced survival rate compared to wild-type flies (p<0.0001); Imd and Toll pathway deficient flies RelishE20 and spätzlerm7, respectively, were used as controls. x-axis: Time post-infection in days; y-axis: Percentage of living flies. Data were analyzed using Log rank test and values are pooled data from three independent experiments. (PDF) [file ppat.1004067.s004.pdf]
